# Association between cardiovascular disease risk, regional brain age gap, and cognition in healthy adults

**DOI:** 10.3389/fnagi.2025.1611847

**Published:** 2025-09-18

**Authors:** Sriya Pallapothu, Roger D. Newman-Norlund, Nicholas Riccardi, Raghav Pallapothu, Pranesh Rajesh Kannan, Leonardo Bonilha, Julius Fridriksson, Chris Rorden

**Affiliations:** ^1^Department of Psychology, McCausland College of Arts and Sciences, University of South Carolina, Columbia, SC, United States; ^2^Department of Communication Sciences & Disorders, Arnold School of Public Health, University of South Carolina, Columbia, SC, United States; ^3^Department of Neurology, School of Medicine Columbia, Columbia, SC, United States

**Keywords:** aging, brain, cardiovascular diseases, risk factors, cognition, magnetic resonance imaging

## Abstract

**Background:**

Cardiovascular disease (CVD) and its associated risk factors accelerate neurodegeneration and cognitive decline. This study examined relationships between CVD risk, cognition, and Brain Age Gap (BAG)—the difference between MRI-predicted brain age and chronological age. While prior research has linked CVD risk factors to global (i.e., “whole-brain”) BAG, we extend these findings by examining region-specific associations, offering more spatially precise insights into brain aging across the cortex.

**Methods:**

Cross-sectional data from 187 participants in the University of South Carolina’s Aging Brain Cohort (ABC) were analyzed. T1-weighted MRI scans were processed with *volBrain*, an automated brain volumetrics pipeline, to calculate global and regional BAG. CVD risk was assessed using the QRISK3 calculator, which provides a 10-year CVD risk percentage and Heart Age value. The Heart Age Gap (HAG) was calculated as Heart Age minus chronological age. Cognitive function was assessed using the Montreal Cognitive Assessment (MoCA). Six data-driven brain aging factors were identified, and participant-level BAG scores for each factor were analyzed. Spearman correlations examined associations between CVD risk metrics, regional BAG factors, and cognition, controlling for age and sex.

**Results:**

10-year CVD risk and HAG were significantly correlated with global BAG (*p* < 0.001), even after adjusting for covariates. The BAGs of Factors 3–6 showed significant positive correlations with 10-year CVD risk and HAG, indicating region-specific vulnerability. Total MoCA was negatively associated with the BAGs of Factors 4–6. In addition, the Language Index was negatively correlated with the BAGs of Factors 1, 4, and 5, while the Executive Index was negatively associated with Factor 5’s BAG. No CVD risk—cognition associations remained significant after adjusting for age.

**Conclusion:**

CVD risk is associated with global and regional brain aging, with specific cortical regions demonstrating greater vulnerability to CVD risk burden than others. These findings highlight the added value of regional BAG analyses, which reveal heterogeneity in aging patterns not captured by global estimates alone and may clarify vascular contributions to brain aging.

## Introduction

Cardiovascular disease (CVD) is the leading cause of death in America (Martin et al., 2025) and in the world (World Health Organization, 2024). It is estimated that the burden of CVD in America will grow in the next few decades (Kazi et al., 2024), which is concerning given that CVD influences not only vascular health but also brain health. CVD and its risk factors are associated with structural changes in the brain, such as increased white matter hyperintensities (WMHs) (Hannan et al., 2025; Jiang et al., 2023; Debette et al., 2011; Jeerakathil et al., 2004), reductions in overall brain volume (Debette et al., 2011; Beauchet et al., 2013; Zhang et al., 2022), and with neurodegenerative conditions like vascular dementia and Alzheimer’s Disease (AD) (Justin et al., 2013; Saeed et al., 2023). Cognitive function has also been linked with cardiovascular health. Premature CVD has been associated with worse cognition in midlife and a greater rate of cognitive decline (Jiang et al., 2023), and CVD risk has been negatively associated with global cognition, executive function (Dregan et al., 2013), and immediate and delayed memory performance (Wei et al., 2023).

As evidence continues to accumulate linking cardiovascular health to brain health and cognitive performance, recent advances in machine learning have enabled the estimation of ‘brain age’ based on T1-weighted structural MRI scans (Franke and Gaser, 2019). Brain age is a single number reflecting the biological age of an individual’s brain, which may differ from their chronological age. Underlying the concept of brain age is the idea that structural and functional changes accumulate across the lifespan. By modeling these age-related trajectories, brain age provides a measure of biological brain integrity, allowing researchers to quantify deviations that may signal disease processes (Franke and Gaser, 2019). The Brain Age Gap (BAG), found by subtracting an individual’s chronological age from their brain age, can suggest rates of brain aging (Franke and Gaser, 2019). A positive BAG is indicative of accelerated aging and has been linked with conditions like schizophrenia (Ballester et al., 2023), depression (Han et al., 2021), progressive mild cognitive impairment, AD (Franke and Gaser, 2019), and longer post-stroke recovery times (Liew et al., 2023). Like other measures of brain health, global (i.e., “whole-brain”) BAG is also heavily influenced by CVD risk factors. Elevated systolic blood pressure (Beck et al., 2022), diabetes, pre-diabetes (Dove et al., 2024), and smoking (Linli et al., 2022) have all been associated with higher global BAG scores. Additionally, in populations with mild cognitive impairment and AD, global BAG has been associated with poor cognition (Franke and Gaser, 2019).

Recent studies have suggested that cerebrovascular risk factors appear to influence BAG in a region-specific manner. Global BAG estimates, while useful for summarizing overall brain health, are too coarse to capture region-specific patterns of neurodegeneration that are critical in aging and disease. A Mendelian randomization study found that elevated diastolic blood pressure was selectively associated with accelerated white matter aging, with effects varying by sex and age (Feng et al., 2023). Another study by Kolbeinsson et al. (2020) found that cardiometabolic risk factors were associated with global BAG and that a few brain regions (e.g., cerebellum, hippocampus, amygdala, and insular cortex) were especially predictive of that estimate, but they did not compute region-specific BAG values or examine how CVD risk relates to regional variability in brain aging. These findings support the idea that certain brain areas may be particularly sensitive to cardiovascular burden. However, they also highlight the need for explicitly modeling BAG at the regional level, rather than inferring it from global models. Prior work on regional vulnerability to aging also supports this approach. A review by Pandya and Patani (2021) reported that regions that are often implicated in neurodegenerative diseases, such as the hippocampus, are more susceptible to accelerated aging and neuronal loss compared to other regions of the brain. Although the studies detailed in the review primarily inferred regional susceptibility to aging by examining gray and white matter volume (Pandya and Patani, 2021), regional BAG metrics offer a refined approach to evaluating region-specific brain aging and may help identify how certain regions are related to peripheral health (Riccardi et al., 2025a; Riccardi et al., 2025b). Surprisingly, no study has yet explored the relationship between regional BAG and CVD risk considering the known connection between CVD risk factors and global brain health.

Additionally, although the association between global BAG and cognition is well-established in clinical populations (Franke and Gaser, 2019), the relationship between BAG and cognition in healthy populations is less clear. Some studies report associations with cognitive decline and domain-specific deficits (Elliott et al., 2021; Boyle et al., 2021), while others show no clear relationship with global cognition (Wrigglesworth et al., 2022). These mixed findings underscore the need to explore how BAG, particularly at the regional level, relates to cognition in non-clinical samples. Though our group has previously shown a relationship between regional BAG and global cognition in a healthy cohort (Riccardi et al., 2025a), domain-specific studies are necessary to further elucidate the relationship between brain aging and distinct cognitive functions.

We sought to examine the relationship between CVD risk, regional BAG, and cognition. Data were obtained from the University of South Carolina’s Aging Brain Cohort (ABC), which provides data on healthy brain aging in adults in South Carolina. The unique dataset includes neuroimaging and lifestyle data, as well as survey-based health information and physiological measurements (Newman-Norlund et al., 2021). Based on prior research, we hypothesized that a regional BAG approach would provide greater insight than a global measure, with certain brain regions being more influenced by CVD risk than others, and that regional variations in BAG would differentially relate to cognitive domains (Feng et al., 2023; Kolbeinsson et al., 2020; Pandya and Patani, 2021; Riccardi et al., 2025a; Riccardi et al., 2025b). Ultimately, findings from this study will enhance the current understanding of the effects of CVD risk on regional brain health which will benefit future clinical applications and therapeutic treatments.

## Methods

### Participants

Data were drawn from the University of South Carolina’s Aging Brain Cohort Study Repository (ABC@USC), an ongoing cross-sectional study of aging which started in 2019. Participants were neurologically healthy and community-dwelling adults (ages 20–80), all of whom were native English speakers. Participants were excluded if they had a diagnosed psychiatric condition (ex: schizophrenia), BMI greater than 42 kg/m^2^, diagnosed neurodegenerative disease, previous history of stroke, acute or chronic conditions that hindered their participation, or any severe illnesses (ex: cancer). Individuals were recruited using a stratified sampling strategy to reflect South Carolina’s diversity in age, gender, race, and socioeconomic status, with income determined by the Hollingshead Index. Recruitment occurred via community outreach (e.g., flyers, brochures, local events, media releases) and online platforms (study website, social media). Interested individuals completed an online eligibility survey, were screened by study staff, and provided written informed consent prior to participation. The Aging Brain Cohort Study was approved by the Institutional Review Board, and full study procedures, including recruitment, screening, and consent, are described in Newman-Norlund et al. (2021).

**Figure 1:**
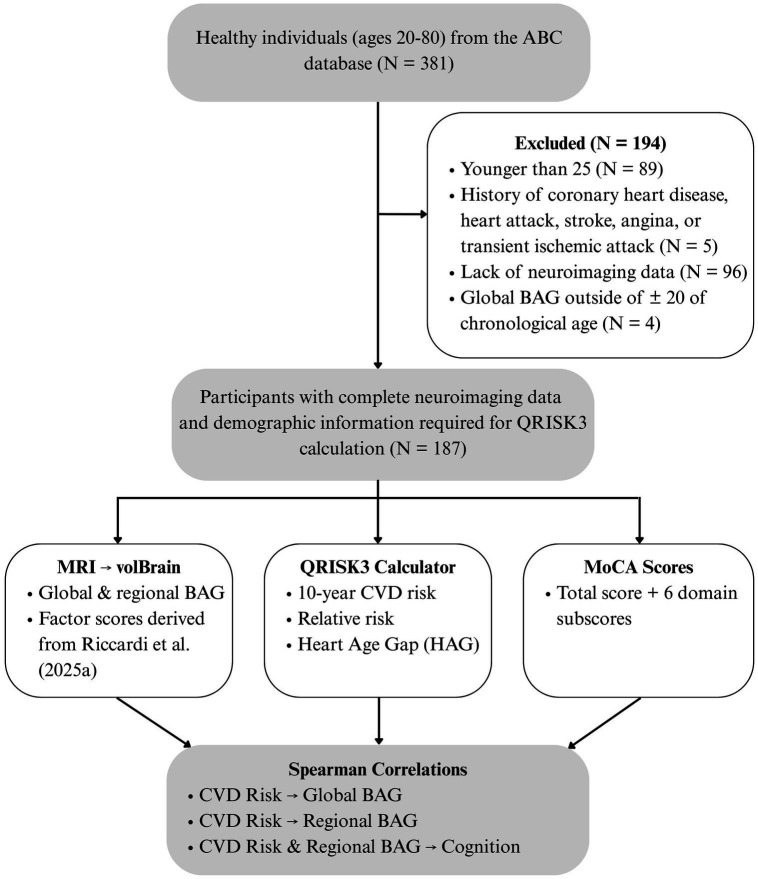
Study methodology overview. Participants were recruited from the Aging Brain Cohort and underwent MRI scans and clinical assessments. Brain age was estimated via volBrain; CVD risk was calculated using QRISK3; and cognitive function was measured via the MoCA. Spearman correlations were performed to assess associations between CVD risk, global and regional BAG, and cognition.

The QRISK3 calculator was used to calculate CVD risk measures^1^ (see *QRISK3* section below). Since the QRISK3 calculator is only valid for individuals between the ages of 25–84 without a history of coronary heart disease, heart attack, angina, stroke, or transient ischemic attack (Hippisley-Cox et al., 2017; Cic, 2024), participants younger than 25 years of age or participants with a clinical history of the above conditions were excluded. Out of a total of 381 available participants, 5 participants were excluded because of the presence of heart attack, transient ischemic attack, or coronary heart disease, and 89 individuals were excluded because they were less than 25 years of age, reducing the sample size to N = 287.96 participants were excluded because they did not have neuroimaging data, and an additional four participants who had a global BAG outside of ±20 of their chronological age were considered outliers and were excluded from all analyses. Ultimately, complete brain imaging (including T1-w MRI images) and demographic information required for the QRISK3 calculator were available for a total of 187 individuals. A total of 169 participants had sufficient demographic data for calculation of QRISK3-based Heart Age (see *QRISK3* section below). See Figure 1 for an illustration chart on study methodology.

### MRI data collection

High-resolution T1-weighted structural MRI scans were acquired for all participants at the McCausland Center for Brain Imaging (Prisma Health Heart Hospital) using a Siemens Prisma Fit 3 T scanner equipped with a 20-channel head coil. A 3D MPRAGE sequence was used with the following parameters: repetition time (TR) = 2,530 ms; echo times (TE) = 1.44, 2.90, 4.36, 5.82, and 7.28 ms; inversion time (TI) = 1,100 ms; flip angle (FA) = 8°; voxel size = 1.0 × 1.0 × 1.0 mm^3^; and matrix size = 256 × 256 × 192 voxels. Foam padding was used to minimize head motion.

### QRISK3

CVD risk metrics were estimated with the QRISK3 model. QRISK3 was created by medical professionals at the National Health Service in the UK and developed with a cohort of primary care patients aged 25–84 years old. QRISK3 predicts an individual’s chance of developing cardiovascular disease—which the model defines as coronary heart disease (including heart attack), ischemic stroke, and transient ischemic attack—within the next decade. The QRISK3 calculator provides composite scores based on multiple factors, including demographics, clinical conditions, and several CVD risk factors like blood pressure, BMI, cholesterol, smoking, and diabetes (Hippisley-Cox et al., 2017). In fact, in a study of 566 individuals with Type 2 diabetes, the QRISK3 model was found to have better predictive ability of CVD than the Framingham Risk Score (Mu et al., 2022), an older model of predicting an individual’s CVD risk in the next 10 years (Jahangiry et al., 2017).

To calculate CVD metrics for each participant, we entered relevant details into the online QRISK3 calculator (see text footnote 1). This included demographic data like age, sex, and ethnicity, as well as physiological measurements like height, weight, systolic blood pressure, and the standard deviation of the 2 most recent systolic blood pressure readings. Other variables included clinical conditions like chronic kidney disease, atrial fibrillation, migraines, diabetes (type 1, type 2, or none) and smoking status (non-smoker, former smoker, and light, moderate, or heavy smoker), and medications like atypical antipsychotics, regular corticosteroid tablets, and blood pressure treatments. The calculator provided the following information for each participant: (1) 10-year CVD risk score, which is an individual’s risk of developing a stroke or heart attack in the next decade, (2) the 10-year CVD risk score of a healthy individual without any clinical risk markers and the same ethnicity, sex, and age as the participant. The healthy individual is assumed to have a cholesterol ratio of 4.0, BMI of 25, and systolic blood pressure of 125 (Cic, 2024). (3) Relative Risk, which is a participant’s 10-year CVD risk score divided by the 10-year score of a healthy individual, and (4) Healthy Heart Age, which is the age at which the participant’s 10-year CVD risk score is achieved by a healthy individual of the same sex and ethnicity (Cic, 2024). Since heart age is a measure of the biological age of an individual’s heart (Lindow et al., 2023), the Heart Age Gap (HAG) was calculated for participants by subtracting their chronological age from their Healthy Heart Age. Previous studies have found that HAG was associated with an increased risk of heart failure, ischemic heart disease, diabetes, and hypertension (Lindow et al., 2023).

When completing the initial ABC demographics questionnaire, participants self-identified as White, Black/African American, Asian, Native Hawaiian/Pacific Islander, American Indian/Alaska Native, and Other. To calculate QRISK3 metrics, individuals must be classified as one of the following ethnicities specified by the calculator: White, Black African, Black Caribbean, Indian, Pakistani, Chinese, Bangladeshi, Other Asian, or Other Ethnic Group. All participants who had identified as Black/African American in the ABC questionnaire were classified as Black African for the QRISK3 calculator, as opposed to Black Caribbean. By examining the languages participants spoke in addition to English, individuals who had self-identified as Asian in the questionnaire were further classified as Indian, Pakistani, Chinese, Bangladeshi, or Other Asian for the QRISK3 calculator. If a participant did not speak another language in addition to English, they were classified as Other Asian. All participants who had identified as Native Hawaiian/Pacific Islander or American Indian/Alaska Native in the questionnaire were classified as Other Ethnic Group for the QRISK3 calculation. Additionally, biracial participants had their QRISK3 scores calculated separately for each identified ethnicity. Then, their two QRISK3 scores were averaged to produce a composite score.

For smoking status, participants who had smoked fewer than 5 packs of cigarettes in their lifetime were classified as non-smokers. Participants who had smoked at least 5 packs in their lifetime but were no longer currently smoking were considered former smokers. Participants who had smoked at least 5 packs in their lifetime and were still smoking were current smokers. Since we did not have data on how many cigarettes each participant was smoking per day, all current smokers were classified as light smokers.

The QRISK3 calculator considers whether an individual has atrial fibrillation, which includes atrial flutter, paroxysmal atrial fibrillation, and atrial fibrillation for the purpose of the calculation (Hippisley-Cox et al., 2017). Although the ABC study did not have data on atrial fibrillation specifically, participants in the study indicated whether they had an irregular heart rhythm. An irregular heart rhythm can include conditions like atrial fibrillation, ventricular fibrillation, atrial flutter, paroxysmal supraventricular tachycardia, and ventricular tachycardia, though atrial fibrillation is the most common cause of irregular heart rhythms (National Heart, Lung, and Blood Institute, 2022). To accurately reflect participants’ clinical risk factors, individuals were only classified with atrial fibrillation if they (1) indicated an irregular heart rhythm, and (2) were taking medications commonly used to treat atrial fibrillation, atrial flutter, or paroxysmal atrial fibrillation.

We assessed whether participants were receiving blood pressure treatment based on their medication lists. If a participant self-reported elevated blood pressure and their medication list included a common antihypertensive drug, the participant was classified as taking blood pressure treatment. If a participant was taking a common blood pressure medication for other conditions (ex: tachycardia) and did not indicate elevated blood pressure, the individual was not labeled as taking blood pressure treatment. However, if a participant did not self-report high blood pressure but was taking a common blood pressure medication without specifying the reason, the participant was classified as receiving blood pressure treatment.

Because certain data were not collected as part of the ABC study, we were unable to submit the following variables to the calculator: the participant’s cholesterol/HDL ratio, a United Kingdom postcode, knowledge of a diagnosis of or treatment for erectile dysfunction, and the presence of rheumatoid arthritis, systemic lupus erythematosus, angina or heart attack in a first degree relative less than 60 years of age, and severe mental illness (includes schizophrenia, bipolar disorder, moderate to severe depression, and psychosis) (Hippisley-Cox et al., 2017; Cic, 2024).

### Brain age calculation

Brain age estimation was performed using the BrainStructureAges pipeline of *volBrain*, an online, validated, and fully automated brain MRI analysis platform designed to extract quantitative features from T1-weighted MRI scans (Manjón and Coupé, 2016; Nguyen et al., 2024; Coupé et al., 2020; de Senneville et al., 2020). The volBrain framework combines multi-atlas segmentation with advanced deep learning–based pipelines to achieve accurate structural parcellation and age prediction (Manjón and Coupé, 2016). The BrainStructureAges module specifically estimates both global and regional brain ages using T1-weighted MRI data as input.

For each participant, *volBrain* utilized T1-weighted structural MRI data to estimate regional brain age. Preprocessing steps, including denoising, inhomogeneity correction, affine registration to the MNI152 space, intensity standardization, and intracranial cavity extraction were applied to each T1w image. Next, the preprocessed images were downscaled by a factor of 2, from the MNI space of 181 × 217 × 181 voxels at 1 mm^3^ to 91 × 109 × 91 voxels (Nguyen et al., 2024). Overlapping 3D subvolumes of the same size (32 × 48 × 32 voxels) were extracted from the images. U-Nets were used to predict the age of each voxel within these subvolumes, and a 3D voxel-wise brain age map (size of 91 × 109 × 91 voxels) was created based on the outputs for each voxel. For voxels included in multiple overlapping subvolumes, predictions were averaged to generate a single brain age value per voxel. Before calculating regional brain ages, the 3D age map was upscaled back to the size of the original image. Then, a segmentation mask created by AssemblyNet was used to parcellate the brain into 132 anatomical regions, including both cortical and subcortical structures. The mean age of the voxels in each region was computed and became that region’s brain age. Additionally, regional age-bias correction was applied via linear regression following the method described in Smith et al. (2019) and Nguyen et al. (2024). To estimate chronological age, a feature vector of the 132 regional brain ages was input into a support vector regression (SVR) model. SVRs were trained using a 10-fold cross-validation approach on 2,887 T1-weighted scans across 8 publicly available datasets. Each SVR fold predicted chronological age, and the outputs from the 10 SVR models were averaged to generate a final chronological age prediction per participant. Model performance was tested using 29,831 scans from the ABIDE II dataset (younger adults) and UK Biobank (older adults). For the younger population, *volBrain*’s method had a mean absolute error (MAE) of 1.88 years and R^2^ of 0.91, while the older population had a MAE of 3.83 years and R^2^ of 0.62. Importantly, *volBrain’*s method provided lower MAE values and higher R^2^ values compared to other state-of-the-art methods for brain age estimation in younger and older populations (Nguyen et al., 2024).

During U-Net training, all voxels within the intracranial cavity were assigned the subject’s chronological age, while non-brain voxels were set to zero. The training set was split 80%/20% for training and validation. Training was optimized using MAE as the loss function and stochastic gradient descent (SGD) with a batch size of 8. Early stopping was applied after 20 epochs without improvement in validation loss (Nguyen et al., 2024). The first U-Net was trained from scratch, while subsequent U-Nets used neighbor transfer learning and were initialized by using the weights of the preceding U-Net. Additionally, the training and validation data were recombined and re-split to train each new U-Net (Nguyen et al., 2024; Coupé et al., 2020). AssemblyNet is the segmentation engine underpinning the regional labeling, utilizing a framework of 3D convolutional neural networks (CNNs) to achieve 3D whole brain segmentation from MRI data. It consists of two assemblies, each containing 125 3D U-Nets that each process a different subvolume. The first assembly provides coarse segmentation at 2 × 2 × 2 mm^3^ resolution, while the second refines this to 1 × 1 × 1 mm^3^. Final predictions from each assembly are generated by majority voting across all 125 U-Nets (Coupé et al., 2020).

For each participant, global and regional BAG values were computed by subtracting chronological age from the estimated brain age (Franke and Gaser, 2019). A positive BAG indicates accelerated brain aging relative to chronological age, while a negative BAG suggests decelerated aging. This metric provides insight into structural brain health and has been linked to neurodegenerative diseases, cognitive performance, and overall neurological resilience (Franke and Gaser, 2019).

### MoCA scores

Participants in the study were administered the Montreal Cognitive Assessment (MoCA) to serve as a measure of cognition function. The MoCA is a 10-min test utilizing a battery of easily administered cognitive tasks, such as memory recall, drawing, fluency, attention, sequencing, verbal, and number-based tasks (Nasreddine et al., 2005). A participant’s level of functioning is measured in six cognitive domains, including memory, executive function, attention, language, visuospatial, and orientation (Wood et al., 2020). The MoCA is scored out of 30 points, and participants with a MoCA score greater than or equal to 26 are considered normal (Nasreddine et al., 2005). Additionally, domain-specific index scores can be calculated for each of the cognitive domains for a more specific analysis of cognition subscores (Wood et al., 2020).

### Statistical analyses

A total of 187 participants were included in the final analysis, and the demographic and clinical characteristics of the sample are presented in Table 1. To evaluate the performance of the *volBrain* model in our dataset, we calculated the coefficient of determination (R^2^) and MAE between chronological age and predicted global brain age. These metrics were computed for the full sample and separately for a healthy subsample. The healthy subsample was limited to participants with 10-year CVD risk scores below the sample median (≤5.90%) and excluded individuals with a history of diabetes, smoking, atrial fibrillation, chronic kidney disease, migraines, and antihypertensive or atypical antipsychotic medication use.

**Table 1:** Summary of participant risk profiles and clinical data.

Category	*N*	Mean (SD)	Min	Max	Range
10-year CVD risk (%)	187	8.25 (8.37)	0.10	36.40	36.30
Relative risk	187	1.36 (0.63)	0.40	4.60	4.20
Healthy heart age	169	59.87 (15.17)	30.00	84.00	54.00
HAG	169	3.62 (4.10)	−4.00	17.00	21.00
BAG	187	−1.85 (5.47)	−18.71	16.16	34.87
Age	187	53.63 (15.71)	25.00	79.00	54.00
Height (cm)	186	168.73 (9.67)	147.32	198.12	50.80
Weight (kg)	186	78.24 (16.85)	45.36	144.70	99.34
Systolic BP (mmHg)	183	125.43 (16.77)	92.00	191.00	99.00

Category	*N*	Details
Sex	187	Female (134), Male (53)
Ethnicity	187	W (155), BA (22), Other (10)
Highest level of education completed	186	HS (15), S (5), C (76), GS (87), Other (3)
Economic status	181	High (73), Medium (93), Low (15)
Smoker	187	NS (135), FS (41), LS (11)
Diabetes	187	Type 1 (1), Type 2 (14), None (172)
CKD	187	Yes (1), No (186)
AF	187	Yes (9), No (178)
BPT	187	Yes (41), No (146)
Migraine	187	Yes (39), No (148)
AAP	187	Yes (3), No (184)
RCT	187	Yes (0), No (187)

Ethnicity: W, Caucasian; BA, Black African; Other, Includes all other ethnicities. Other Ethnicity makeup: 3 Indian, 1 Chinese, 1 Bangladeshi, 2 Other Asian, 3 Biracial. Level of Education Completed: HS, High School; S, at least 1 year of college/specialized training; C, College/University; GS, Graduate School. Smoker: NS, Nonsmoker; FS, Former Smoker; LS, Light Smoker. Diabetes: None, No diabetes; Type 1, Type 1 diabetes; Type 2, Type 2 diabetes. Additional Conditions: CKD, Chronic Kidney Disease; AF, Atrial Fibrillation; BPT, Blood Pressure Treatment; AAP, Atypical Antipsychotics; RCT, Regular Corticosteroid Tablets.

To evaluate the relationship between CVD risk and global brain aging, we performed positive one-tailed Spearman correlations between CVD 10-year risk, Relative Risk, Heart Age Gap (HAG), and global Brain Age Gap (BAG) (Table 2), based on the *a priori* hypothesis that increased CVD risk would be associated with accelerated brain aging, which is supported by prior research (Beck et al., 2022; Dove et al., 2024; Linli et al., 2022). To examine associations between CVD risk and regional brain aging patterns, we leveraged a six-factor parcellation of the brain previously defined by Riccardi et al. (2025a). This study applied an exploratory factor analysis to 104 regional brain age estimates derived from *volBrain*. The factor analysis identified six distinct spatial patterns of coordinated brain aging that reflect underlying neurobiological hierarchies, and the observed factors replicated in independent datasets. We chose to use these predefined factors to increase the biological interpretability and reproducibility of our analyses, as well as to ensure consistency with prior work. For regional analyses, we used participant-level scores on these six factors, which were calculated in the same way as the earlier study by our group (Riccardi et al., 2025a). These scores reflect the degree to which each participant expresses the spatial brain aging pattern defined by each factor. Positive one-tailed tests of Spearman’s rank correlations were then performed between 10-year CVD risk, HAG, and the six regional BAG values (Table 3). The relationships between 10-year CVD risk, HAG, regional BAG, and MoCA scores were also assessed with Spearman’s correlations (Tables 4, 5). Negative one-tailed tests were performed because we hypothesized that increased BAG would be associated with worse cognitive function based on earlier studies (Elliott et al., 2021; Wrigglesworth et al., 2022; Boyle et al., 2021; Franke and Gaser, 2019).

**Table 2:** Relationships between CVD risk metrics and global BAG.

Variable	Statistic	10-year CVD risk	Relative risk	HAG
Relative risk	*n*	187	—	
	Spearman’s rho	**0.687** ^***^	—	
	p_FDR_	3.69 × 10^−27^		
	*p*-value	1.846 × 10^−27^	—	
HAG	*n*	169	169	—
	Spearman’s rho	**0.767** ^***^	**0.97** ^***^	—
	p_FDR_	2.12 × 10^−33^	4.56 × 10^−103^	
	*p*-value	7.072 × 10^−34^	7.602 × 10^−104^	—
BAG	*n*	187	187	169
	Spearman’s rho	**0.285** ^***^	**0.206** ^**^	**0.267** ^***^
	p_FDR_	6.47 × 10^−5^	0.002	2.91×10^−4^
	*p*-value	4.313 × 10^−5^	0.002	2.429 × 10^−4^

All tests were one-tailed for positive correlations and conditioned on sex and age. Significant correlations that survived omnibus FDR corrections are bolded. ^*^p < 0.05, ^**^p < 0.01, ^***^p < 0.001 (based on unadjusted *p*-values).

**Table 3:** Relationships between CVD risk metrics and regional BAGs.

Variable	Statistic	10-year CVD risk	HAG
Factor 1	*n*	187	169
	Spearman’s rho	0.112	0.016
	p_FDR_	0.110	0.505
	*p*-value	0.064	0.421
Factor 2	*n*	187	169
	Spearman’s rho	0.021	0.039
	p_FDR_	0.505	0.461
	*p*-value	0.388	0.307
Factor 3	*n*	187	169
	Spearman’s rho	**0.204** ^**^	**0.238** ^***^
	p_FDR_	0.007	0.003
	*p*-value	0.003	9.670 × 10^−4^
Factor 4	*n*	187	169
	Spearman’s rho	**0.163** ^*^	**0.251** ^***^
	p_FDR_	0.026	0.003
	*p*-value	0.013	5.274 × 10^−4^
Factor 5	*n*	187	169
	Spearman’s rho	**0.233** ^***^	**0.31** ^***^
	p_FDR_	0.003	2.70 × 10^−4^
	*p*-value	7.197 × 10^−4^	2.246 × 10^−5^
Factor 6	*n*	187	169
	Spearman’s rho	−0.144	−0.031
	p_FDR_	0.975	0.711
	*p*-value	0.975	0.652

All tests were one-tailed for positive correlations and conditioned on sex and age. Significant correlations that survived omnibus FDR corrections are bolded. ^*^p < 0.05, ^**^p < 0.01, ^***^p < 0.001 (based on unadjusted *p*-values).

**Table 4:** Relationships between CVD risk metrics and MoCA scores.

Variable	Statistic	10-year CVD risk	HAG
Total MoCA	*n*	187	169
	Spearman’s rho	−0.024	−0.082
	*p*-value	0.375	0.147
Memory index	*n*	187	169
	Spearman’s rho	0.017	−0.075
	*p*-value	0.589	0.166
Executive index	*n*	187	169
	Spearman’s rho	−0.021	−0.068
	*p*-value	0.386	0.19
Attention and concentration index	*n*	187	169
	Spearman’s rho	−0.05	−0.082
	*p*-value	0.249	0.145
Language index	*n*	187	169
	Spearman’s rho	−0.011	−0.038
	*p*-value	0.441	0.314
Visuospatial index	*n*	187	169
	Spearman’s rho	−0.053	−0.117
	*p*-value	0.236	0.067
Orientation index	*n*	187	169
	Spearman’s rho	0.003	0.04
	*p*-value	0.515	0.698

All tests were one-tailed for negative correlations and conditioned on sex and age. No correlations reached nominal significance, so multiple comparison correction was not applied.

**Table 5:** Relationships between regional BAGs and MoCA scores.

Variable	Statistic	Factor 1	Factor 2	Factor 3	Factor 4	Factor 5	Factor 6
Total MoCA	*n*	187	187	187	187	187	187
	Spearman’s rho	−5.062 × 10^−4^	−0.053	−0.074	−0.149*	−0.157*	**−0.153** ^*^
	p_FDR_	0.497	0.284	0.237	0.044	0.044	0.044
	*p*-value	0.497	0.237	0.158	0.022	0.016	0.019
Memory index	*n*	187	187	187	187	187	187
	Spearman’s rho	0.133	−0.033	−0.037	−0.075	−0.058	−0.136^*^
	p_FDR_	0.965	0.396	0.396	0.396	0.396	0.192
	*p*-value	0.965	0.33	0.307	0.157	0.217	0.032
Executive index	*n*	187	187	187	187	187	187
	Spearman’s rho	−0.05	−0.013	−0.095	−0.093	**−0.21** ^**^	−0.026
	p_FDR_	0.375	0.430	0.206	0.206	0.012	0.430
	*p*-value	0.250	0.430	0.099	0.103	0.002	0.362
Attention and concentration index	*n*	187	187	187	187	187	187
	Spearman’s rho	−0.038	−0.005	−0.065	−0.051	−0.141^*^	−0.077
	p_FDR_	0.365	0.475	0.365	0.365	0.168	0.365
	*p*-value	0.304	0.475	0.190	0.244	0.028	0.149
Language index	*n*	187	187	187	187	187	187
	Spearman’s rho	**−0.169** ^*^	0.052	−0.022	**−0.202** ^**^	**−0.168** ^*^	−0.055
	p_FDR_	0.022	0.758	0.458	0.018	0.022	0.341
	*p*-value	0.011	0.758	0.382	0.003	0.011	0.227
Visuospatial index	*n*	187	187	187	187	187	187
	Spearman’s rho	−0.052	−0.007	−0.107	−0.054	−0.104	−0.058
	p_FDR_	0.288	0.460	0.237	0.288	0.237	0.288
	*p*-value	0.240	0.460	0.073	0.234	0.079	0.217
Orientation index	*n*	187	187	187	187	187	187
	Spearman’s rho	0.034	−0.034	0.032	0.011	0.05	−0.175^**^
	p_FDR_	0.748	0.748	0.748	0.748	0.748	0.054
	*p*-value	0.676	0.321	0.67	0.559	0.748	0.009

All tests were one-tailed for negative correlations and conditioned on sex and age. FDR correction was applied within each MoCA subdomain individually. Significant correlations that survived corrections are bolded. ^*^p < 0.05, ^**^p < 0.01, ^***^p < 0.001 (based on unadjusted *p*-values).

**Figure 2:**
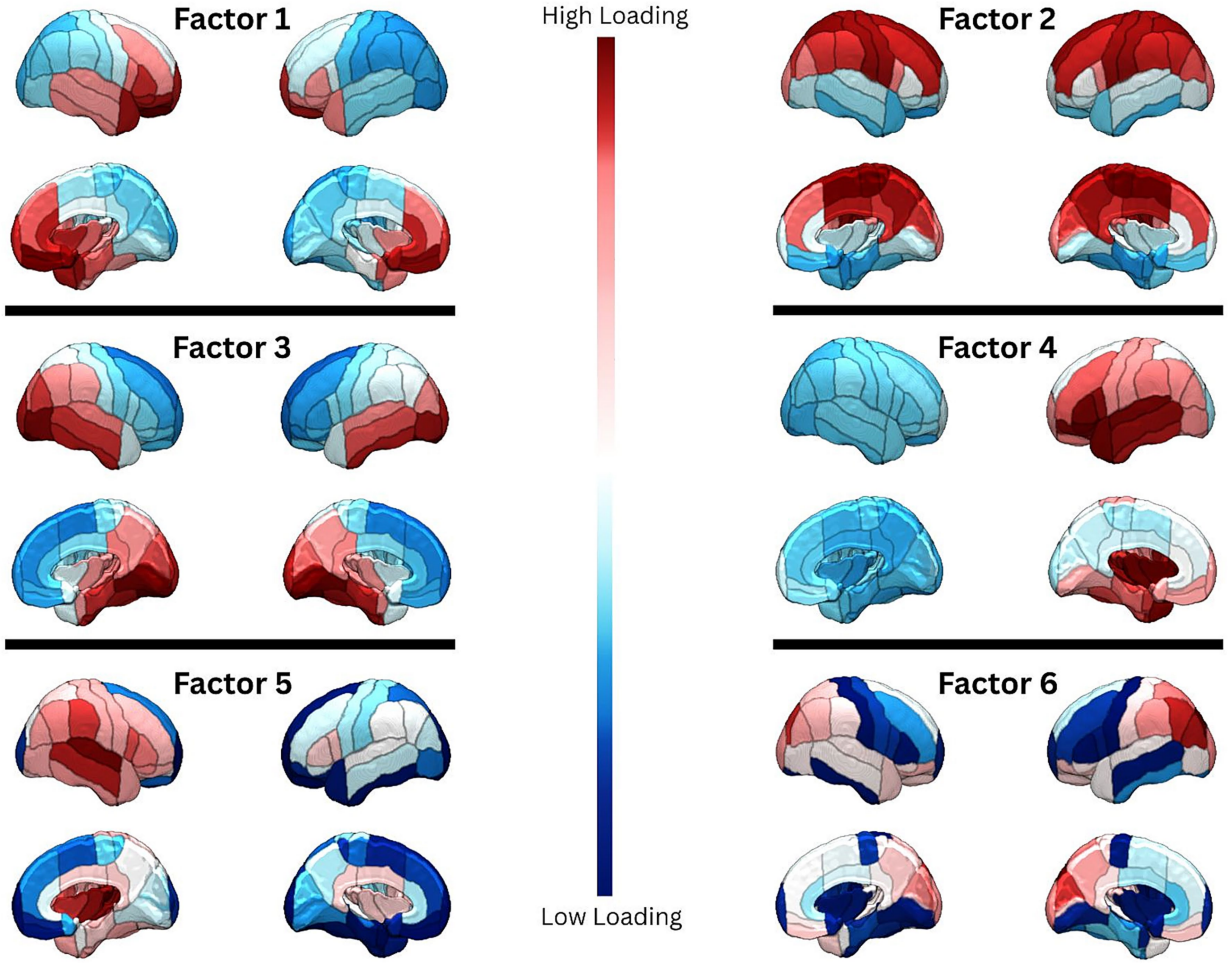
Spatial distributions of the six factors. 3D surface renderings of the six factors are displayed in lateral and medial views for each hemisphere. Dark red indicates regions with higher factor loadings, while dark blue indicates regions with lower factor loadings.

All statistical analyses were conducted using version 0.19.1 of JASP, an open-source statistical platform built on the R programming language (JASP Team, 2024). For our analyses in Tables 2–5 we controlled for sex and age, and false discovery rate (FDR) correction was applied using the Benjamini–Hochberg procedure. For Table 5, FDR corrections were conducted within each MoCA subdomain individually, as the subdomains are related but represent distinct cognitive domains. For the other analyses, an omnibus FDR correction was applied across all comparisons. Spatial distributions of the 6 factors (Figure 2) were created in MRIcroGL, a free neuroimaging visualization software (Rorden and Brett, 2000). Additionally, we conducted a supplementary exploratory analysis assessing correlations between CVD risk metrics and the original 104 cortical regional brain age values from *volBrain* (Supplementary Table 1). Given the large number of comparisons in this table, two-tailed tests were utilized to minimize the risk of inflated false positives. These were uncorrected and exploratory supplementary findings that were not conditioned on sex or age, and should therefore be interpreted with caution.

## Results

The average age of participants in the study was 53.63 ± 15.71 years. The average Healthy Heart Age was slightly higher, at 59.87 ± 15.17 years. 72% of the participants were female, and 83% of participants identified as white, 12% identified as black, and 5% identified as other ethnic groups. The average 10-year CVD risk score for participants was 8.25 ± 8.37%, while the average Relative Risk was 1.36 ± 0.63. The average BAG was −1.85 ± 5.47, and the average HAG was 3.62 ± 4.10 (Table 1).

For the full sample (*N* = 187), the correlation between chronological age and predicted global brain age provided a R^2^ of 0.898 and MAE of 4.41 years. These metrics indicate that *volBrain* predictions closely track chronological age in our dataset, supporting the validity of using BAG as a biologically meaningful marker of individual differences in brain aging. Additionally, in our healthy subsample (*N* = 50), the MAE was 5.34 years and the R^2^ was 0.818, supporting the reliability of *volBrain* predictions in generally healthy adults.

Global BAG had a significant and positive relationship with 10-year CVD risk [Spearman: r (187) = 0.285, p_FDR_ < 0.001], Relative Risk [Spearman: r (187) = 0.206, p_FDR_ = 0.002], and HAG [Spearman: r (169) = 0.267, p_FDR_ < 0.001]. Additionally, 10-year CVD risk, Relative Risk, and HAG were all significantly positively correlated with each other. Notably, Relative Risk and HAG showed very high collinearity [Spearman: r (169) = 0.97, p_FDR_ < 0.001], suggesting possible overlap in the information they convey. Given this redundancy, and the stronger association between HAG and global BAG, we chose to retain only 10-year CVD risk and HAG for further analyses (Table 2).

As shown in Figure 2, Factor 1 primarily includes frontal regions. Factor 2 encompasses dorsal areas; Factor 3 covers ventral regions; Factor 4 is lateralized to the left hemisphere and spans the left frontotemporal cortex; Factor 5 is lateralized to the right hemisphere and involves the right temporoparietal cortex; and Factor 6 reflects bilateral lateral occipital and parietal regions. HAG was significantly positively correlated with Factors 3–5 (r range = 0.238–0.31, all p_FDR_’s < 0.01), and 10-year CVD risk was also significantly positively correlated with Factors 3–5 (r range = 0.163–0.233, all p_FDR_’s < 0.05) (Table 3).

The relationship between CVD risk metrics, regional BAG, and cognition was also assessed. Initial analyses revealed significant negative correlations between HAG and the Memory Index, as well as between 10-year CVD risk and cognitive outcomes (total MoCA as well as the Memory and Visuospatial indices), after correcting for multiple comparisons; however, these associations did not remain significant after controlling for age and sex (Table 4), suggesting that the observed effects were primarily driven by age-related variance rather than CVD risk. Additionally, significant relationships were found between our regional BAG factors and cognitive subscores. The BAGs of Factors 4–6 were significantly negatively associated with total MoCA score (p_FDR_ < 0.05), with Factor 5’s BAG also showing a negative correlation with the Executive Index (p_FDR_ = 0.012). In addition, the BAGs of Factors 1, 4, and 5 were negatively associated with the Language Index (p_FDR_ < 0.05), while the correlation between Factor 6’s BAG and the Orientation Index approached significance (p_FDR_ = 0.054) (Table 5).

## Discussion

### Overview of study findings

This study examined the relationship between CVD risk (QRISK3 10-year risk and HAG), cognition (MoCA total and MoCA subscores), and regional BAG in six previously identified networks. We hypothesized that CVD risk metrics, previously found to be related to global BAG, would disproportionately affect the BAG of some regions, and that regional BAG measures would show distinct associations with different cognitive domains. Our results were generally consistent with these hypotheses. Specifically, regional BAG was significantly positively correlated with 10-year CVD risk and HAG for Factors 3, 4, and 5, but not for Factors 1, 2, or 6. These findings suggest that while CVD risk influences brain aging, its effects are not uniform across the entire brain; instead, certain regions may be more vulnerable to cardiovascular related aging than others. Additionally, our regional BAG metrics were associated with specific MoCA subdomains, supporting the idea that region-specific patterns of brain aging may relate to distinct cognitive functions. These results support the utility of a regional BAG approach over a global one when investigating the relationship between CVD risk, brain health, and cognition. However, contrary to our expectations, 10-year CVD risk and HAG were not significantly associated with cognitive performance.

Our study confirmed that global BAG was significantly positively correlated with 10-year CVD risk and HAG (Table 2), which is consistent with prior literature examining CVD risk factors and brain health (Beck et al., 2022; Dove et al., 2024; Linli et al., 2022). Additionally, our factor-specific analysis revealed regional variations in brain aging in relation to 10-year CVD risk and HAG (Table 3). Notably, all analyses with BAG were conditioned on age and sex, suggesting that CVD risk is associated with global and regional brain aging beyond the effects of age and sex alone. Importantly, the brain structure age estimates were corrected for age bias using the method of Smith et al. (2019), as implemented in the *volBrain* pipeline (Nguyen et al., 2024), reducing the influence of regression-to-the-mean artifacts in BAG.

### Association between CVD risk metrics and regional BAG factors

The six BAG factors used in our analyses were derived from a prior study that found distinct patterns of regional brain aging which reflected underlying neurobiological hierarchies with heterogeneous spatial distributions. These factors were aligned with established neuroanatomical gradients, including aerobic glycolysis, gene expression, and anatomical hierarchy, and have been shown to map onto established cortical organization principles such as the sensory–association (S-A) axis (Riccardi et al., 2025a; Sydnor et al., 2021). These spatial patterns replicated across independent cohorts and explained cognitive and sensorimotor performance better than global brain age, underscoring their biological and behavioral relevance (Riccardi et al., 2025a).

Our analyses showed that the BAGs of Factors 3–5 were positively associated with both 10-year CVD risk and HAG, implying these factors’ unique spatial patterns may better capture vascularly sensitive aging processes. For instance, Factor 3 is composed of ventral and inferior regions, such as the posterior occipital-temporal regions, hippocampus, and fusiform gyrus. These regions can be sensitive to vascular health, hypoperfusion (Gonzalez et al., 2015; Beason-Held et al., 2007), and metabolic stress (Tomasi et al., 2013; Palombit et al., 2022; Zhao and Flavin, 2000), perhaps contributing to higher regional BAG values. Cardiovascular metrics like elevated diastolic blood pressure have been shown to predict thinning in areas like the occipital pole (Gonzalez et al., 2015), while longitudinal data suggests that hypertensive individuals have greater decreases in cerebral blood flow in posterior occipital regions and occipitotemporal regions as compared to healthy controls (Beason-Held et al., 2007). These findings align with our observation that regions within Factor 3 may be especially susceptible to vascular changes associated with CVD risk.

Mechanistically, chronic hypoperfusion can deplete the brain of glucose and lead to oxidative stress and inflammation (Rajeev et al., 2023; Park et al., 2019), which may ultimately result in cell death, the development of white matter lesions, and the breakdown of the blood–brain barrier (Rajeev et al., 2023). Cerebral hypoperfusion is associated with several CVD risk factors (Meyer et al., 2000), making this a plausible mechanism behind our results. Furthermore, certain areas in Factor 3 like the medial occipital regions and hippocampus have a high metabolic demand (Tomasi et al., 2013; Palombit et al., 2022) and display increased sensitivity to oxygen and glucose deprivation (Zhao and Flavin, 2000), potentially making them especially vulnerable in the context of vascular compromise. Individuals with suboptimal metabolic status have been shown to display greater brain aging in the parahippocampal gyri and left hippocampus and fusiform (Haas et al., 2024), further supporting the idea that ventral and posterior brain regions in our study may be vulnerable to accelerated aging via multiple systemic pathways. Our findings link this vulnerability to CVD risk—an aggregate measure of vascular and select metabolic factors—whereas Haas et al. (2024) report similar effects in relation to metabolic status alone, suggesting that vascular and metabolic processes may independently or jointly contribute to aging in these areas.

The positive association of the BAGs of Factors 4 and 5 with 10-year CVD risk and HAG may reflect similar mechanisms. Factor 4 is lateralized to the left hemisphere, while Factor 5 is lateralized to the right hemisphere. However, both factors encompass areas traditionally supplied by the middle cerebral artery (MCA), which is the most common major cerebral artery affected by acute stroke (Nogles and Galuska, 2023; Ng et al., 2007) and large artery atherosclerosis (Balambighai et al., 2025). Notably, the MCA also displays a less robust system of collateral circulation. Primary collateral circulation is supplied by the Circle of Willis, while secondary collateral circulation is supplied by leptomeningeal vessels—pre-existing vessels that connect the distal branches of the major cerebral arteries and are activated during chronic hypoperfusion (Liebeskind, 2003; Tariq and Khatri, 2008). Critically, the MCA is relatively under-supplied by the Circle of Willis compared to other major arteries (Zhao et al., 2024), increasing its reliance on secondary collaterals such as leptomeningeal vessels (Zhao et al., 2024; Akamatsu et al., 2015). Risk factors like diabetes (Akamatsu et al., 2015; Li et al., 2024), metabolic syndrome (Menon et al., 2013), hypertension (Li et al., 2024), and age (Menon et al., 2013; Li et al., 2024) may contribute to poor secondary collateral circulation, suggesting that individuals with higher CVD risk may have reduced capacity for compensatory blood flow in the MCA arterial territory. Higher CVD risk scores have also been associated with lower blood velocity (Perdomo et al., 2019) and higher pulsatility (Pase et al., 2012) in the MCA, further supporting a potential vascular mechanism behind the observed associations with Factors 4 and 5.

Furthermore, the proximity of regions in Factors 4 and 5 to the watershed zone between the middle and posterior cerebral artery (PCA) territories may also be related to the observed accelerated brain aging in these areas. Watershed zones lie at the distal margins of arterial territories and are especially susceptible to ischemia due to low perfusion pressure and long, branching arterial supply (Mangla et al., 2011; Dogariu et al., 2023). Infarcts in cortical watersheds are often due to microemboli from atheromatous plaque (Mangla et al., 2011; Dogariu et al., 2023), suggesting these areas are sensitive to the effects of vascular factors and atherosclerosis. Thus, the spatial overlap of Factors 4 and 5 with vulnerable vascular territories provides a biologically plausible explanation for their association with CVD risk.

Although Factors 1 and 2 also capture a few regions that are relatively metabolically active (Tomasi et al., 2013; Palombit et al., 2022), BAG in neither of these factors was significantly associated with 10-year CVD risk or HAG. This may reflect greater vascular resilience in these regions. Factor 1 primarily encompasses medial frontal and subcortical areas supplied by the anterior cerebral artery (ACA), which benefits from robust primary collateral support via the anterior communicating artery (Liebeskind, 2003). This well-developed collateral system may help preserve perfusion and protect against vascular insufficiency, potentially explaining the lack of association with 10-year CVD risk and HAG. Factor 2, while including some regions supplied by the middle cerebral artery (MCA), is situated closer to the ACA–MCA watershed zone. Notably, the ACA–MCA watershed region has a higher density of leptomeningeal vessels than the MCA–PCA watershed zone (Liebeskind, 2003), suggesting that Factor 2 may receive more redundant or mixed vascular supply. This may account for the presence of cardiovascular-related effects in Factors 4 and 5 but absence of cardiovascular-related effects in Factor 2. Additionally, although we focused primarily on hypoperfusion as a candidate mechanism for regional brain aging, other pathophysiological factors may contribute. These include blood–brain barrier dysfunction (Knox et al., 2022), altered cellular composition and gene expression (Wang et al., 2025), and mitochondrial dysfunction (Bartman et al., 2024). Future studies could integrate vascular imaging, transcriptomics, or metabolic profiling to further explore these contributors.

### Association between cognitive subscores, CVD risk, and regional BAG factors

Unadjusted analyses revealed that 10-year CVD risk was negatively correlated with total MoCA as well as the Memory and Visuospatial Indices, while HAG was significantly correlated with the Memory Index. The significant relationship between 10-year CVD risk and total MoCA and the Memory Index is consistent with prior research supporting the idea that CVD risk significantly influences global cognition and memory (Song et al., 2020). However, in our sample, this association did not hold when controlling for age (Table 4). This suggests that the relationship between CVD risk metrics and cognition may have been primarily driven by age rather than CVD risk factors in our sample. It is also possible that our limited sample size impaired our ability to detect subtle effects of CVD risk on cognition after adjusting for age.

Interestingly, our analyses did reveal that regional BAG factors were significantly negatively associated with select MoCA subdomains (Table 5). While prior studies have linked higher global BAG to poor general cognition in healthy populations (Elliott et al., 2021; Boyle et al., 2021), our findings extend this work by identifying specific regional brain aging patterns related to both global and domain-specific cognition. The BAGs of Factors 4–6 were significantly negatively associated with total MoCA scores (Table 5), in line with an earlier study showing similar associations for these factors (Riccardi et al., 2025a). In domain-specific analyses, Factor 5’s BAG was negatively associated with the Executive Index, while the BAGs of Factors 1, 4, and 5 were negatively linked to the Language Index (Table 5). These results partially align with Tan et al. (2025), who reported that BAG mediated the relationship between cognitive impairment risk factors and global cognition, executive function, and language in individuals with high cerebrovascular disease burden. Notably, Tan et al. examined global BAG, whereas our study focuses on regional patterns of brain aging, which may reveal associations obscured at the global level.

The utility of a regional approach is supported by prior work. For example, a recent study found that, in participants with cerebral small vessel disease, regional BAG was a stronger mediator of cognitive outcomes than global BAG (Lee et al., 2022). Similarly, in patients with left-hemisphere stroke, higher BAG in nonlesioned left domain-general regions predicted greater aphasia severity (Busby et al., 2024), underscoring the clinical utility of targeted regional measures. This latter finding may help explain our observed association between Factor 4’s BAG and the Language Index, as Factor 4 is highly lateralized to the left hemisphere (Figure 2). Collectively, these patterns suggest that specific brain networks underlie distinct cognitive domains and that their vulnerability to accelerated aging can be missed when examining global BAG alone. Our findings, though preliminary, highlight the potential biological relevance of these relationships and the need for future studies with larger samples and hypothesis-driven designs to clarify the links between regional brain aging and cognitive performance.

### Limitations

Our data were cross-sectional, but longitudinal analyses could allow for stronger causal claims about the relationship between CVD risk and brain structure. Though participant-level factor scores in our sample were calculated using the same approach as the original study that derived the factors (Riccardi et al., 2025a), our sample included 20 more ABC participants. This modest increase in sample size may contribute to minor variations in results. While we lacked access to all the information requested by the QRISK3 calculator — which could have affected the 10-year CVD risk, Relative Risk, and HAG scores calculated for each participant — inclusion of these additional variables would likely have had minimal effects, as the primary drivers of the calculation were included (see *QRISK3* section of the Methods). Furthermore, although the QRISK3 calculator is used internationally and has been verified in international groups (Hippisley-Cox et al., 2017), it was originally designed specifically for use in the UK population (Cic, 2024). It is possible that differences in healthcare access, diet, genetics, and lifestyle could have impacted the accuracy scores generated by the QRISK3 calculator for our South Carolina sample. Additionally, the QRISK3 calculator did not account for female-specific risk factors, such as gestational diabetes, preeclampsia, or early menopause, that increase a woman’s risk for CVD (Appelman et al., 2015); thus, the calculator may not have fully captured women’s risk of cardiovascular events, which could have affected the observed relationships with regional BAG. Finally, factors beyond CVD risk likely influence brain structure across the adult lifespan (Busby et al., 2022; Vidal-Pineiro et al., 2021; de Lange et al., 2021). Since we did not analyze the role of other potential risk factors on regional brain aging, our findings may not fully capture the complex interplay of factors affecting regional brain health.

### Future directions

To our knowledge, the relationship between CVD risk and regional BAG has not been previously studied. While the current investigations provide some insights into this relationship, future larger scale studies are necessary to evaluate and validate some of the less robust findings reported here. Additionally, future studies should analyze the role of the vasculature in relation to regional BAG and CVD risk. Vascular factors are implicated in both cardiovascular aging and cognitive decline (King et al., 2023), and they may explain the link between CVD risk and BAG. Analysis of regional BAG using arterial atlases, such as those produced by Liu et al. (2023) could shed light on the mechanisms connecting CVD risk to brain health, which could inform future therapeutic treatments. Studies examining the impact of other risk factors, such as inflammation, small vessel disease, or female-specific risk factors, may also provide a more comprehensive understanding of regional brain aging. Finally, future research should investigate whether metrics that integrate both CVD risk and brain health measures are superior for predicting mortality when compared to measures that focus solely on CVD risk or brain health. Cardiovascular health and brain aging are likely influenced by similar risk factors and may share an underlying etiology, so considering both may provide a more holistic assessment of an individual’s health.

## Final conclusion

CVD risk influences brain aging in a heterogenous matter with certain networks being disproportionately affected by CVD risk burden. Importantly, we demonstrate, for the first time, the added value of using a regional BAG approach in studying the relationship between brain health and CVD risk. Future studies might examine regional BAGs of areas/networks defined by vascular parcellations of the brain to further elucidate the relationship between cardiovascular health and brain health.

## Data Availability

The raw data supporting the conclusions of this article will be made available by the authors upon request, without undue reservation.

## Footnotes

^1^ https://qrisk.org/index.php

## Supplementary material

SUPPLEMENTARY TABLE 1 Relationships between CVD risk metrics and BAGs of 104 regions.
